# Impact of Covid-19 on risk of severe maternal morbidity

**DOI:** 10.1186/s13054-023-04584-6

**Published:** 2023-09-05

**Authors:** Nathalie Auger, U. Vivian Ukah, Shu Qin Wei, Jessica Healy-Profitós, Ernest Lo, Natalie Dayan

**Affiliations:** 1 University of Montreal Hospital Research Centre, Montreal, QC Canada; 2 Institut national de santé publique du Québec, Montreal, QC Canada; 3 Department of Epidemiology, Biostatistics, and Occupational Health, McGill University, Montreal, QC Canada; 4 Department of Social and Preventive Medicine, School of Public Health, University of Montreal, Montreal, QC Canada; 5 HealthPartners Institute, Pregnancy and Child Health Research Center, Bloomington, MN USA; 6 Department of Obstetrics and Gynecology, University of Montreal, Montreal, QC Canada; 7 Department of Medicine, McGill University, Montreal, QC Canada

**Keywords:** Covid-19, Hemorrhage, Pregnancy, Renal failure, SARS-CoV-2, Severe maternal morbidity

## Abstract

**Background:**

We examined the risk of severe life-threatening morbidity in pregnant patients with Covid-19 infection.

**Methods:**

We conducted a population-based study of 162,576 pregnancies between March 2020 and March 2022 in Quebec, Canada. The main exposure was Covid-19 infection, including the severity, period of infection (antepartum, peripartum), and circulating variant (wildtype, alpha, delta, omicron). The outcome was severe maternal morbidity during pregnancy up to 42 days postpartum. We estimated risk ratios (RR) and 95% confidence intervals (CI) for the association between Covid-19 infection and severe maternal morbidity using adjusted log-binomial regression models.

**Results:**

Covid-19 infection was associated with twice the risk of severe maternal morbidity compared with no infection (RR 2.02, 95% CI 1.76–2.31). Risks were elevated for acute renal failure (RR 3.01, 95% CI 1.79–5.06), embolism, shock, sepsis, and disseminated intravascular coagulation (RR 1.35, 95% CI 0.95–1.93), and severe hemorrhage (RR 1.49, 95% CI 1.09–2.04). Severe antepartum (RR 13.60, 95% CI 10.72–17.26) and peripartum infections (RR 20.93, 95% CI 17.11–25.60) were strongly associated with severe maternal morbidity. Mild antepartum infections also increased the risk, but to a lesser magnitude (RR 3.43, 95% CI 2.42–4.86). Risk of severe maternal morbidity was around 3 times greater during circulation of wildtype and the alpha and delta variants, but only 1.2 times greater during omicron.

**Conclusions:**

Covid-19 infection during pregnancy increases risk of life-threatening maternal morbidity, including renal, embolic, and hemorrhagic complications. Severe Covid-19 infection with any variant in the antepartum or peripartum periods all increase the risk of severe maternal morbidity.

**Supplementary Information:**

The online version contains supplementary material available at 10.1186/s13054-023-04584-6.

## Background

Covid-19 infection during pregnancy is associated with preterm birth and stillbirth [1, 2], but less is known about the risk of severe maternal morbidity. Severe maternal morbidity comprises life-threatening events such as thromboembolism, severe hemorrhage, and sepsis, many of which may naturally complicate Covid-19 infection [3–5]. A meta-analysis of 18 studies found that Covid-19 tripled the risk of intensive care unit admission, need for respiratory support, and use of mechanical ventilation in pregnant women [6]. While these studies provide important information on the need for critical care, many cases may be due to respiratory complications that are not necessarily related to pregnancy or severe maternal morbidity. Very little is known about the types of life-threatening morbidities that women with Covid-19 may be at risk of during pregnancy.

The impact of Covid-19 on severe maternal morbidity is poorly understood because prior research has focused on demonstrating that newer SARS-CoV-2 variants are not as dangerous as earlier variants in pregnant women [6–9]. Studies have successfully shown that infections with wildtype, alpha, beta, gamma, and delta are all more harmful than omicron [6–9]. As the attention was on comparing variants, efforts at identifying the specific life-threatening morbidities in SARS-CoV-2 infected women have been rare. Only two studies have addressed this issue, both suggesting that infected pregnant women who require critical care may be at risk of severe obstetrical hemorrhage, venous thromboembolism, and respiratory morbidity [10, 11]. However, other types of severe maternal morbidity were not examined, leaving the underlying effect of Covid-19 in pregnancy largely unknown. We examined how SARS-CoV-2 infection was associated with different types of severe maternal morbidity in a large cohort of pregnant women.

## Methods

### Study population

We performed a population-based cohort study of 162,576 deliveries in Quebec, Canada, between March 1, 2020 and March 31, 2022. We extracted data on the deliveries from the Maintenance and Use of Data for the Study of Hospital Clientele registry, which contains discharge summaries for hospitalizations before, during, and after pregnancy [12]. The data include nearly all pregnancies that led to a live birth or stillbirth in Quebec, as 98% occur in hospital.

The data contain patient diagnoses and procedures during hospitalization, coded using the tenth revision of the International Classification of Diseases (ICD-10) and Canadian Classification of Health Interventions (CCI). We identified any diagnosis of severe maternal morbidity during hospitalizations between conception and 42 days following pregnancy. We also identified any diagnosis of a Covid-19 infection during antepartum and peripartum hospitalizations. We only considered Covid-19 infections that were present before or during admissions at which there was an occurrence of severe maternal morbidity, to ensure that the exposure preceded the outcome. We excluded women with invalid identifiers as we could not capture all clinical events in these patients.

### SARS-CoV-2 infection

The main exposure measure was SARS-CoV-2 infection during antepartum admissions or at delivery. During the study period, hospitals in Quebec systematically screened pregnant women for SARS-CoV-2 infection at admission [13]. In addition, at the time of delivery, hospitals collected data from outpatient charts on past infections that did not require admission [13]. Thus, we were able to identify antepartum Covid-19 infections that required admission prior to delivery, antepartum infections that did not require admission, and peripartum infections at the delivery admission. Infection was confirmed by polymerase chain reaction and identified through ICD-10 codes (Additional file 1: eTable 1).

We further classified SARS-CoV-2 infection by severity, based on symptoms or comorbid conditions during admission (Additional file 1: eTable 1). To identify severe cases, we used the National Institutes of Health’s Covid-19 severity guidelines [14, 15]. Women with moderate to severe Covid-19 symptoms, including pneumonia, bronchitis, lower respiratory tract complications, pulmonary embolism, and other related conditions were classified as having severe Covid-19. Patients with symptoms such as cough and sore throat, or who were asymptomatic, were classified as having mild Covid-19.

We analyzed four time periods based on the dominant circulating strain of SARS-CoV-2, including wildtype (March 1, 2020 to April 3, 2021), alpha (April 4, 2021 to July 31, 2021), delta (August 1 to December 11, 2021), and omicron (December 12, 2021 to March 31, 2022) [16]. These variants accounted for more than 50% of infections in each time period [16].

### Severe maternal morbidity

The main outcome measure was severe maternal morbidity, following the definition of the Public Health Agency of Canada [3]. Severe maternal morbidity was defined as life-threatening conditions during pregnancy or within 42 days postpartum, including severe preeclampsia or eclampsia; severe hemorrhage requiring transfusion or secondary to a coagulation defect; embolism, shock, sepsis, or disseminated intravascular coagulation; acute renal failure; hysterectomy and surgical complications; other rare life-threatening morbidity (cardiomyopathy, cardiac arrest and resuscitation, myocardial infarction, pulmonary edema and heart failure, cardiac complications of anesthesia, cerebrovascular accident, acute fatty liver requiring transfusion, hepatic failure, cerebral edema or coma, complications of anesthesia, status asthmaticus, adult respiratory distress syndrome, acute abdomen, uterine rupture, inverted uterus, sickle cell anemia with crisis, acute psychosis, status epilepticus); admission to an intensive care unit; and assisted ventilation [17]. We identified the timing of severe maternal morbidity (during antepartum admissions, at the delivery hospitalization, during postpartum readmissions) and total number of severe maternal morbidities (1, 2 or more).

### Covariates

We examined factors that could influence the association between Covid-19 infection and severe maternal morbidity, such as maternal age (< 25, 25–34, ≥ 35 years), parity (0, 1, ≥ 2 previous deliveries), comorbidity including preexisting diabetes, hypertension, obesity, dyslipidemia, and tobacco, alcohol, and substance use disorders (yes, no), socioeconomic disadvantage (advantaged, disadvantaged, unspecified), and place of residence (rural, urban, unspecified). Socioeconomic disadvantage was measured using a composite index of the proportion of individuals with low education, unemployment, and low income in the immediate neighborhood [18].

### Statistical analysis

We calculated the rate of severe maternal morbidity per 1000 deliveries for women with and without Covid-19, and estimated risk ratios (RR) and 95% confidence intervals (CI) for the association of SARS-CoV-2 infection with risk of severe maternal morbidity using adjusted log-binomial regression models. Models were adjusted for maternal age, parity, comorbidity, socioeconomic disadvantage, and place of residence. We estimated associations for different types of severe morbidity, as well as according to timing and total number of morbidities. The reference group comprised women without Covid-19 infection during pregnancy.

In sensitivity analyses, we changed the reference group to patients who delivered before the pandemic (January 1, 2017 to February 29, 2020), to determine if the associations could have been attenuated by an effect of pandemic containment measures.

We analyzed the data in SAS v9.4 (SAS Institute Inc., Cary, NC) and used an alpha level of 0.05 with a two-tailed test to determine statistical significance. As we used anonymized data, we obtained an ethics waiver from our research institution.

## Results

Among 162,576 deliveries between March 2020 and March 2022, 3,415 patients (2.1%) had a SARS-CoV-2 infection during pregnancy (Additional file 1: eTable 2). Patients with SARS-CoV-2 infections had higher rates of severe maternal morbidity than uninfected patients (6.1% vs. 3.0%). Covid-19 infection was more frequent in women aged ≥ 35 years and women who were multiparous or socioeconomically disadvantaged.

Covid-19 was associated with an increased risk of severe maternal morbidity (Table 1). Any Covid-19 infection during pregnancy was associated with 2.02 times the risk of severe maternal morbidity compared with no infection (95% CI 1.76–2.31). Associations were most prominent for acute renal failure (RR 3.01, 95% CI 1.79–5.06), assisted ventilation (RR 12.78, 95% CI 8.32–19.62), and intensive care unit admission (RR 6.16, 95% CI 5.04–7.54). Covid-19 was strongly associated with severe maternal morbidity during antepartum admissions (RR 5.25, 95% CI 3.94–7.01), although an elevated risk was also present with severe maternal morbidity during the delivery admission (RR 1.78, 95% CI 1.52–2.10) and during postpartum readmissions (RR 1.43, 95% CI 0.98–2.07).

**Table 1:** Association of Covid-19 infection with severe maternal morbidity

	No. deliveries		Rate of severe maternal morbidity per 1000 deliveries		Risk ratio (95% CI)^a^ Covid-19 vs. no infection
	Covid-19 (N = 3415)	No infection (N = 159161)	Covid-19	No infection	
Severe maternal morbidity					
Any	208	4774	60.9	30.0	2.02 (1.76, 2.31)
Severe preeclampsia, eclampsia	38	1313	11.1	8.2	1.30 (0.94, 1.81)
Severe hemorrhage	40	1281	11.7	8.0	1.49 (1.09, 2.04)
Embolism, shock, sepsis, disseminated intravascular coagulation	32	1089	9.4	6.8	1.35 (0.95, 1.93)
Acute renal failure	16	232	4.7	1.5	3.01 (1.79, 5.06)
Hysterectomy, surgical complications	13	567	3.8	3.6	1.04 (0.60, 1.80)
Intensive care admission	106	786	31.0	4.9	6.16 (5.04, 7.54)
Assisted ventilation	28	96	8.2	0.6	12.78 (8.32, 19.62)
Other life-threatening morbidity	33	689	9.7	4.3	2.14 (1.50, 3.06)
Timing of severe maternal morbidity					
Antepartum	51	452	14.9	2.8	5.26 (3.94, 7.01)
Peripartum	128	3366	37.5	21.1	1.78 (1.50, 2.12)
Postpartum	29	956	8.5	6.0	1.43 (0.99, 2.08)
Total number of morbidities					
1	146	3887	42.8	24.4	1.78 (1.51, 2.09)
≥ 2	62	887	18.2	5.6	3.23 (2.49, 4.18)

^a^Adjusted for maternal age, parity, comorbidity, socioeconomic disadvantage, and place of residence

The timing of infection during pregnancy influenced the risk of severe maternal morbidity (Table 2). Severe Covid-19 during antepartum (RR 13.60, 95% CI 10.72–17.26) and delivery admissions (RR 20.93, 95% CI 17.11–25.60) was associated with 13 to 20 times the risk of severe maternal morbidity compared with no infection. Mild Covid-19 during antepartum (RR 3.43, 95% CI 2.42–4.86) and delivery admissions (RR 1.34, 95% CI 1.06–1.70) was less strongly associated with severe maternal morbidity. In contrast, antepartum infections that never required hospitalization were not associated with severe maternal morbidity (RR 0.92, 95% CI 0.66–1.28).

**Table 2:** Association of severity of Covid-19 infection and circulating variant with risk of severe maternal morbidity

	No. deliveries with severe maternal morbidity	No. deliveries	Rate per 1000 deliveries	Risk ratio (95% CI)^a^
Timing and severity of infection				
Severe peripartum Covid-19	31	42	738.1	20.93 (17.11, 25.60)
Severe antepartum Covid-19	43	103	417.5	13.60 (10.72, 17.26)
Mild peripartum Covid-19	67	1708	39.2	1.34 (1.06, 1.70)
Mild antepartum Covid-19 with admission	29	272	106.6	3.43 (2.42, 4.86)
Mild antepartum Covid-19 without admission	38	1290	29.5	0.92 (0.66, 1.28)
No infection	4774	159161	30.0	Reference
Dominant variant				
Wildtype	58	663	87.5	2.88 (2.26, 3.68)
Alpha	42	400	105.0	3.39 (2.53, 4.55)
Delta	33	369	89.4	2.92 (2.08, 4.10)
Omicron	75	1983	37.8	1.27 (1.01, 1.59)
No infection	4774	159161	30.0	Reference

^a^Adjusted for maternal age, parity, comorbidity, socioeconomic disadvantage, and place of residence

Patients with severe Covid-19 infection during the delivery admission were at risk of most types of severe maternal morbidity (Table 3). Risks of assisted ventilation (RR 490.17, 95% CI 297.54–807.50), intensive care admission (RR 104.43, 95% CI 82.00–133.01), acute renal failure (RR 41.26, 95% CI 13.76–123.72), embolism, shock, sepsis, or disseminated intravascular coagulation (RR 6.83, 95% CI 1.74–26.87), and severe hemorrhage (RR 11.25, 95% CI 4.33–29.27) were elevated. Mild Covid-19 infections at delivery were nevertheless associated with renal failure and intensive care unit admission, although at lower magnitudes.

**Table 3:** Association of Covid-19 infection during delivery admission with risk of severe maternal morbidity

	Prevalence of severe maternal morbidity per 1000 deliveries			Risk ratio (95% CI)^a^	
	Severe Covid-19	Mild Covid-19	No infection	Severe Covid-19 at delivery	Mild Covid-19 at delivery
Severe maternal morbidity					
Any	738.1	39.2	30.0	20.89 (17.06, 25.59)	1.34 (1.06, 1.70)
Severe preeclampsia, eclampsia	23.8	12.9	8.2	2.51 (0.39, 16.37)	1.62 (1.07, 2.46)
Severe hemorrhage	95.2	12.9	8.0	11.25 (4.33, 29.27)	1.66 (1.09, 2.51)
Embolism, shock, sepsis, disseminated intravascular coagulation	47.6	7.0	6.8	6.83 (1.74, 26.87)	1.05 (0.60, 1.85)
Acute renal failure	71.4	4.1	1.5	41.26 (13.76, 123.72)	2.87 (1.35, 6.07)
Hysterectomy, surgical complications	71.4	2.3	3.6	14.41 (4.61, 45.03)	0.66 (0.25, 1.78)
Intensive care admission	690.5	11.1	4.9	104.43 (82.00, 133.01)	2.27 (1.44, 3.59)
Assisted ventilation	381.0	1.8	0.6	490.17 (297.54, 807.50)	2.88 (0.92, 9.08)
Other life-threatening morbidity	190.5	4.7	4.3	34.02 (17.51, 66.12)	1.06 (0.51, 2.18)
Timing of severe maternal morbidity					
Peripartum	714.3	29.9	21.1	28.94 (23.21, 36.10)	1.45 (1.11, 1.91)
Postpartum	23.8	9.4	6.0	13.00 (1.97, 85.59)	1.62 (0.99, 2.64)
Total number of morbidities					
1	309.5	26.3	24.4	19.08 (13.13, 27.71)	1.12 (0.84, 1.49)
≥ 2	428.6	12.9	5.6	88.85 (64.20, 122.97)	2.38 (1.56, 3.62)

^a^Association for severe or mild infection relative to no infection, adjusted for maternal age, parity, comorbidity, socioeconomic disadvantage, and place of residence

Patients with antepartum Covid-19 infections were also at risk of severe maternal morbidity (Table 4). Compared with no infection, severe antepartum infections were associated with use of assisted ventilation (RR 85.19, 95% CI 37.08–195.74), intensive care admission (RR 70.96, 95% CI 53.95–93.34), acute renal failure (RR 12.70, 95% CI 3.22–50.13), and embolism, shock, sepsis, or disseminated intravascular coagulation (RR 8.42, 95% CI 3.87–18.34). Mild infections during antepartum hospitalizations were also associated with most of these outcomes, but to a lesser extent. Antepartum infections that did not require hospitalization were not associated with most outcomes.

**Table 4:** Association of antepartum Covid-19 infection with risk of severe maternal morbidity

	Risk ratio (95% CI)^a^		
	Severe Covid-19 during antepartum admission	Mild Covid-19 during antepartum admission^b^	Antepartum Covid-19 without admission
Severe maternal morbidity			
Any	13.62 (10.73, 17.28)	3.43 (2.42, 4.86)	0.92 (0.66, 1.28)
Severe preeclampsia, eclampsia	1.19 (0.17, 8.32)	2.09 (0.86, 5.05)	0.67 (0.32, 1.40)
Severe hemorrhage	3.57 (1.19, 10.77)	1.83 (0.70, 4.81)	0.69 (0.33, 1.45)
Embolism, shock, sepsis, disseminated intravascular coagulation	8.42 (3.87, 18.34)	1.60 (0.52, 4.90)	0.93 (0.46, 1.85)
Acute renal failure	12.70 (3.22, 50.13)	2.39 (0.34, 16.88)	1.06 (0.26, 4.24)
Hysterectomy, surgical complications	2.27 (0.33, 15.79)	0.96 (0.14, 6.85)	0.83 (0.31, 2.23)
Intensive care admission	70.96 (53.95, 93.34)	9.68 (5.73, 16.34)	0.79 (0.33, 1.90)
Assisted ventilation	85.19 (37.08, 195.74)	5.69 (0.79, 40.90)	2.55 (0.63, 10.34)
Other life-threatening morbidity	10.86 (4.63, 25.50)	4.94 (2.25, 10.84)	1.09 (0.50, 2.39)
Timing of severe maternal morbidity			
Antepartum	120.71 (91.35, 159.50)	17.36 (10.34, 29.16)	-
Peripartum	3.58 (1.53, 8.37)	2.10 (1.21, 3.67)	1.04 (0.72, 1.50)
Postpartum	2.57 (0.37, 17.77)	1.96 (0.64, 6.00)	0.91 (0.44, 1.91)
Total number of morbidities			
1	14.26 (10.75, 18.93)	3.42 (2.30, 5.08)	0.97 (0.68, 1.38)
≥ 2	23.40 (12.99, 42.17)	3.98 (1.80, 8.79)	0.69 (0.29, 1.67)

^a^Association for severe or mild infection relative to no infection, adjusted for maternal age, parity, comorbidity, socioeconomic disadvantage, and place of residence

^b^67% of patients hospitalized with mild antenatal Covid-19 were admitted for obstetric reasons. Associations persisted when we removed these potentially incidental cases

The alpha variant, followed by delta and wildtype, were associated with the greatest risk of severe maternal morbidity (Table 5). These strains were most strongly associated with acute renal failure and other life-threatening morbidity (4 to 6 times greater risk). Associations weakened during omicron, although Covid-19 remained associated with assisted ventilation (RR 7.41, 95% CI 3.75–14.64) and intensive care unit admission (RR 3.03, 95% 2.10–4.36).

**Table 5:** Association of dominant circulating variant with risk of severe maternal morbidity

	Risk ratio (95% CI)^a^			
	Wild type	Alpha	Delta	Omicron
Severe maternal morbidity				
Any	2.88 (2.26, 3.68)	3.39 (2.53, 4.55)	2.92 (2.08, 4.10)	1.27 (1.01, 1.59)
Severe preeclampsia, eclampsia	1.79 (0.97, 3.31)	1.89 (0.85, 4.18)	0.67 (0.17, 2.73)	1.13 (0.71, 1.79)
Severe hemorrhage	1.69 (0.88, 3.23)	1.59 (0.67, 3.80)	1.72 (0.72, 4.10)	1.36 (0.88, 2.08)
Embolism, shock, sepsis, disseminated intravascular coagulation	2.22 (1.20, 4.11)	1.49 (0.56, 3.95)	2.02 (0.85, 4.83)	0.90 (0.51, 1.59)
Acute renal failure	3.96 (1.47, 10.63)	5.16 (1.66, 16.09)	5.61 (1.81, 17.41)	1.75 (0.72, 4.21)
Hysterectomy, surgical complications	1.96 (0.82, 4.70)	2.00 (0.64, 6.25)	–	0.71 (0.29, 1.71)
Intensive care admission	9.33 (6.58, 13.23)	12.20 (8.25, 18.04)	10.90 (7.07, 16.81)	3.03 (2.10, 4.36)
Assisted ventilation	11.92 (4.86, 29.26)	28.27 (13.15, 60.78)	26.23 (11.61, 59.57)	7.41 (3.75, 14.64)
Other life-threatening morbidity	1.68 (0.70, 4.00)	3.99 (1.92, 8.29)	4.19 (1.89, 9.32)	1.53 (0.89, 2.65)
Timing of severe maternal morbidity				
Antepartum	7.61 (4.51, 12.86)	10.95 (6.26, 19.14)	10.32 (5.41, 19.69)	2.48 (1.47, 4.18)
Peripartum	2.88 (2.14, 3.88)	2.90 (1.95, 4.32)	2.14 (1.32, 3.47)	1.12 (0.84, 1.50)
Postpartum	1.07 (0.40, 2.84)	2.25 (0.94, 5.39)	2.37 (0.99, 5.67)	1.22 (0.72, 2.06)
Total number of morbidities				
1	2.64 (1.97, 3.53)	3.10 (2.18, 4.39)	2.63 (1.76, 3.94)	1.07 (0.81, 1.40)
≥ 2	4.45 (2.74, 7.22)	5.30 (2.95, 9.52)	4.49 (2.35, 8.59)	2.20 (1.47, 3.29)

^a^Association for dominant variant relative to no infection, adjusted for maternal age, parity, comorbidity, socioeconomic disadvantage, and place of residence

We obtained similar results when we changed the reference group to patients who delivered before the pandemic.

## Discussion

This population-based cohort study suggests that Covid-19 infection during pregnancy was associated with an increased risk of severe maternal morbidity that stayed relatively consistent over the course of the first two years of the pandemic, up until the arrival of the omicron variant. Risk of severe maternal morbidity was greatest during circulation of wildtype and the alpha and delta variants. The omicron variant remained associated with a greater risk of severe maternal morbidity, but at a lower magnitude. Severe antepartum or peripartum infections were associated with most types of severe maternal morbidity, particularly acute renal failure, severe hemorrhage, and embolism, shock, sepsis, and disseminated intravascular coagulation. Associations were weaker or absent for mild antepartum and peripartum infections. Our findings suggest that pregnant patients with Covid-19 are at risk of several types of severe maternal morbidity, with the dominant circulating variant and infection severity modifying the risk. Renal, hemorrhagic, and embolic complications seem to be the most prevalent.

Severe maternal morbidity includes serious complications during pregnancy, delivery, or within 42 days postpartum that are life-threatening [19]. Although the exact definition varies from study to study, complications such as eclampsia, obstetric hemorrhage, and heart failure are generally included. Yet, studies of the impact of Covid-19 on severe pregnancy outcomes generally focus on measures of critical care, not the underlying morbidities requiring care [2]. A meta-analysis of more than 30 studies found that pregnant women with Covid-19 were around 2.5 times more likely to require intensive care and assisted ventilation than infected nonpregnant women [2]. Risks were 5.4 times greater when infected pregnant women were compared with uninfected pregnant women. However, the investigators could not identify the specific life-threatening conditions that led to critical care. As many indications for critical care may not relate to pregnancy, the extent to which Covid-19 affects serious maternal morbidity remains unclear [20].

Understanding the types of pregnancy complications that are more prevalent in patients with Covid-19 requires use of a validated measure of severe maternal morbidity [10]. Yet, only a handful of studies have examined standardized measures of severe maternal morbidity meant to identify life-threatening outcomes of pregnancy [8, 10, 11, 15]. A multicenter study of 3 129 patients with SARS-CoV-2 found that infection at delivery was associated with twice the risk of any severe maternal morbidity measured as a composite indicator, compared with 12 504 uninfected patients in the U.S. [8]. Early variants were associated with up to seven times the risk of severe respiratory, non-respiratory, and non-hemorrhagic maternal morbidity, while omicron was associated with severe respiratory morbidity only [8]. Other studies have found that Covid-19 infection was associated with 1.5 to 2.5 times the risk of any life-threatening outcome, again measured using a composite score [10, 11, 15]. Only two studies have assessed specific morbidities, finding that obstetrical hemorrhage and venous thromboembolism were the most common severe outcomes in women with Covid-19 [10, 11]. However, the number of morbidities examined was limited in both studies. Using a comprehensive measure of severe maternal morbidity, we were able to demonstrate that pregnant women were at risk of these and several other complications that have not previously been examined.

In our data, some of the strongest associations were with acute renal failure, embolism, shock, sepsis, disseminated intravascular coagulation, and severe hemorrhage. Risk of renal failure was elevated even for mild peripartum Covid-19 infections. Renal injury has been widely reported in nonpregnant patients with Covid-19 [21]. The kidney may be particularly sensitive to Covid-19 during pregnancy, as glomerular filtration rates increase up to 50% by the early second trimester [22]. As a result, superimposed stress or shock due to SARS-CoV-2 infection could more readily lead to renal injury [23]. A U.S. multicenter study found that the severe morbidities most prevalent among women with Covid-19 were renal failure, sepsis, and transfusion, although the relative risk of these outcomes was not measured [8]. Embolic complications may also be prevalent. A study of 43 886 patients from California found that pregnant women with SARS-CoV-2 had three times the risk of venous thromboembolism and greater rates of sepsis [11]. Some of these findings could be explained by placental malperfusion, thrombosis, or inflammation, as pregnant women with Covid-19 tend to be at risk of these disorders [15]. Covid-19 is also associated with coagulopathy [10], which may increase the risk of hemorrhage or disseminated intravascular coagulation [11, 24].

The severity of Covid-19 infection affected the magnitude of association with severe maternal morbidity. We found that severe infections had a greater impact, although mild infections increased the risk of some outcomes, such as renal failure. In the only other study that assessed severity of Covid-19, severe infections were also associated with a greater risk of maternal morbidity [15]. However, the analysis was restricted to pregnancies before December 2020 and did not include a number of complications that are normally considered part of severe maternal morbidity [15]. Disseminated intravascular coagulation has been reported in pregnant women with mild or asymptomatic Covid-19 [24].

Women with severe antepartum or peripartum Covid-19 infections were all at risk of severe maternal morbidity. Antepartum infections may impact outcomes at delivery through vascular malperfusion or other pathological changes in the placenta, as placental dysfunction is known to persist after infection resolves [25]. Patients may also develop placental abnormalities associated with preeclampsia or coagulation defects that increase the risk of renal failure and severe hemorrhage at delivery [24, 26, 27]. Yet, prior studies have paid less attention to antepartum infections. An analysis of seven hospitals from New York found that both antepartum and peripartum infections increased the risk of obstetrical hemorrhage at delivery [10]. Underlying conditions such as obesity and diabetes may exacerbate the risk of Covid-19 complications and severe maternal morbidity [2, 17].

This study has limitations. We used administrative data that may be affected by misclassification of exposures or outcomes that may have attenuated the strength of associations. We did not have information on potential confounders such as ethnicity and number of prenatal visits. However, it is unlikely that residual confounding explains the findings as associations were strong for most types of severe maternal morbidity. We did not have data on maternal vaccination status, although vaccination during pregnancy was not actively promoted in our population until the latter part of 2021 [28]. Part of the reduced risk during omicron may be due to vaccination. It is possible some cases of severe preeclampsia were misdiagnosed owing to an overlap with Covid-19 symptoms. Patients with Covid-19 may also have had different levels of antenatal care. Results for intensive care should be interpreted with caution as women may have been admitted to intensive care units as a safeguard, especially early in the pandemic*.* We could not analyze self-reported Covid-19 infections. We likely did not capture all mild or asymptomatic antepartum cases, which may have affected results for infections not requiring hospitalization. We could not confirm the SARS-CoV-2 strain, however the dominant variant accounted for more than 50% of infections during each time period [8, 16]. Quebec provides universal healthcare, but future research is needed to determine if the findings are generalizable to areas with alternate healthcare systems.

## Conclusions

This study of more than 160000 patients found that Covid-19 infection during pregnancy was associated with several types of severe maternal morbidity, including acute renal failure, severe hemorrhage, impaired coagulation, and embolism. Severe infections drove most of the excess risk, whether infections occurred antenatally or near the time of delivery. All dominant strains of SARS-CoV-2 were associated with severe maternal morbidity, although the risk was not as elevated during omicron. As there appears to be a continued risk of renal, embolic, and hemorrhagic severe maternal morbidity, care providers should remain vigilant and continue promoting vaccination during pregnancy.

### Supplementary Information

**Additional file 1.** eTable 1 and eTable 2.

## Data Availability

The data that support the study findings are available from the Quebec Statistics Institute (https://statistique.quebec.ca/research/#/demarche/etape-par-etapee).
