# Supplementary material for: Impact of Covid-19 on risk of severe maternal morbidity
Source: Crit Care. 2023 Sep 5;27:344. doi: 10.1186/s13054-023-04584-6 (PMC10481463; doi:10.1186/s13054-023-04584-6)
Supplement: Supplementary file 1 — Additional file 1. eTable 1 and eTable 2. [file 13054_2023_4584_MOESM1_ESM.docx]

**ADDITIONAL FILE 1**

**eTable 1. Diagnostic and procedure codes for Covid-19 and severe maternal morbidity**

**eTable 2. Characteristics of patients with and without Covid-19 infection**

**eTable 1** Diagnostic and procedure codes for Covid-19 and severe maternal morbidity

|  | International Classification of Diseases 10th revision | Canadian Classification of Health Interventions |
| --- | --- | --- |
| Covid-19 | U07.1, U07.2, U07.4, U07.5 |  |
| Mild Covid-19 | A09.0, A09.9, G43.0-G43.9, G44.1-G44.8, G93.3, J0.0-J02.9, J04.0-J09, J11.1, J11.8, K29.0, K29.1, K29.6-K29.9, K52.3-K52.9, K59.1, R00.0, R00.2, R00.8, R03.0, R03.1, R05, R06.7, R07.0, R07.3, R07.4, R09.8-R11.3, R13, R50.8-R52.0, R52.9, R53, R56, R59, Z03.8, Z03.9, Z04.8, Z04.9, Z51.9 |  |
| Severe Covid-19 | I26, J11.0, J12-J22, J39.8-J40, J44, J80, J90, J91, J96, J98.0, J98.4, J98.8, J98.9, J99.8, R06.0-R06.5, R06.8, R07.1, R07.2, R09.3, R57.1-R57.9, R65, R91, U04 |  |
| Severe maternal morbidity |  |  |
| Severe preeclampsia, eclampsia | O14.1, O14.2, O15 |  |
| Severe hemorrhage | O44.1, O45.0, O46.0, O67, O72 | 1.LZ.19.HH, U1, A, 1.LZ.19.HH, U1, J, 1.LZ.19.HH, U9, A, 1.LZ.19.HH, U9, J, 1.LZ.19.HM, U1, 1.LZ.19.HM, U9, 1.LZ.35.HA, T7, 1.LZ.35.HH, T7, 1.RM.87.LA, GX, 1.RM.89.LA, 5.MD.60.CB, 5.MD.60.KE, 5.MD.60.RC, 5.MD.60.RD, 5.PC.91.GA, 5.PC.91.GC, 5.PC.91.GD |
| Embolism, shock, sepsis, disseminated intravascular coagulation | D65, O75.1, O75.3, O85, O88, R57, T80.5, T88.6 |  |
| Acute renal failure | N17, N19, N99.0, O90.4, | 1.PZ.21 |
| Hysterectomy, surgical complications^a^ | O75.4 | 1.RM.87.LA, GX, 1.RM.89.LA, 1.LZ.19.HH-U1-A, 1.LZ.19.HH-U1-J, 1.LZ.19.HH-U9-A, 1.LZ.19.HH-U9-J, 1.LZ.19.HM-U1, 1.LZ.19.HM-U9, 1.LZ.35.HAT7, 1.LZ.35.HH-T7, 1.NK.80, 1.NM.80, 5.MD.60.CB, 5.MD.60.KE, 5.MD.60.RC, 5.MD.60.RD, 5.PC.73.JS, 5.PC.80.JH, 5.PC.80.JM, 5.PC.80.JR |
| Other life-threatening morbidity^b^ | B20-B24, D57.0, F23, F53.1, G41, G93.6, I21, I22, I42, I43, I46, I49.0, I50, J45.01, J45.11, J45.81, J45.91, I60-I64, J80, J81, K35, K37, K65, K71, K72, N73.3, N73.5, O14 (2006-2011), O14.1-O14.2 (2012-2019), O22.5, O26.6, O29.0-O29.2, O71.0, O71.1, O74.0, O74.2, O74.3, O87.3, O89.0-O89.2, O90.3, O98.7, R40.2 | 1.HZ.09, 1.HZ.30, 1.LZ.19.HH-U1-A, 1.LZ.19.HH-U1-J, 1.LZ.19.HH-U2-A, 1.LZ.19.HH-U2-J, 1.LZ.19.HH-U9-A, 1.LZ.19.HH-U9-J, 1.LZ.19.HM-U1, 1.LZ.19.HMU2, 1.LZ.19.HM-U9, 1.LZ.35.HA-T7, 1.LZ.35.HH-T7, 1.RM.87.LA-GX, 1.RM.89.LA, 5.MD.60.CB, 5.MD.60.KE, 5.MD.60.RC, 5.MD.60.RD, 5.PC.91.HQ, 5.PC.91.HP |
| Assisted ventilation |  | 1.GZ.31.CA-ND, 1.GZ.31.CR-ND |

^a^Complications of obstetric procedures, repair of bladder, urethra, or intestine, complication requiring transfusion.

^b^Cardiomyopathy, cardiac arrest and resuscitation, myocardial infarction, pulmonary edema and heart failure, cardiac complications of anesthesia, cerebrovascular accident, acute fatty liver requiring transfusion, hepatic failure, cerebral oedema or coma, complications of anaesthesia, status asthmaticus, adult respiratory distress syndrome, acute abdomen, uterine rupture, inverted uterus, sickle cell anaemia with crisis, acute psychosis, status epilepticus.

**eTable 2** Characteristics of patients with and without Covid-19 infection

|  | No. Deliveries (%) | |
| --- | --- | --- |
|  | Covid-19 | No infection |
| Severe maternal morbidity |  |  |
| Yes | 208 (6.1) | 4,774 (3.0) |
| No | 3,207 (93.9) | 154,387 (97.0) |
| Maternal age, years |  |  |
| ≤24 | 415 (12.2) | 17,544 (11.0) |
| 25-34 | 2,154 (63.1) | 106,330 (66.8) |
| ≥35 | 846 (24.8) | 35,287 (22.2) |
| Parity |  |  |
| 0 | 1,553 (45.5) | 77,283 (48.6) |
| 1 | 1,092 (32.0) | 53,705 (33.7) |
| ≥2 | 770 (22.6) | 28,173 (17.7) |
| Maternal comorbidity^c^ |  |  |
| Yes | 473 (13.9) | 20,219 (12.7) |
| No | 2,942 (86.2) | 138,942 (87.3) |
| Socioeconomic disadvantage |  |  |
| Advantaged | 2,345 (68.7) | 117,668 (73.9) |
| Disadvantaged | 840 (24.6) | 31,734 (19.9) |
| Place of residence |  |  |
| Urban | 2,839 (83.1) | 125,624 (78.9) |
| Rural | 468 (13.7) | 29,551 (18.6) |
| Total | 3,415 (100) | 159,161 (100) |
